# Epidemiology and Viral Etiology of the Influenza-Like Illness in Corsica during the 2012–2013 Winter: An Analysis of Several Sentinel Surveillance Systems

**DOI:** 10.1371/journal.pone.0100388

**Published:** 2014-06-24

**Authors:** Laëtitia Minodier, Christophe Arena, Guillaume Heuze, Marc Ruello, Jean Pierre Amoros, Cécile Souty, Laurent Varesi, Alessandra Falchi

**Affiliations:** 1 EA7310, Laboratoire de Virologie, Université de Corse, Institut national de la Santé et de la Recherche Médicale, Corte, France; 2 Observatoire régional de la Santé de Corse, Ajaccio, France; 3 Cellule de l'Institut national de Veille Sanitaire en région, Ajaccio, France; 4 Sorbonne Universités Pierre et Marie Curie, Paris 06, UMRS 1136, Institut Pierre Louis d'Epidémiologie et de Santé Publique, Paris, France; 5 Institut national de la Santé et de la Recherche Médicale, UMRS 1136, Institut Pierre Louis d'Epidémiologie et de Santé Publique, Paris, France; Faculty of Biochemistry Biophysics and Biotechnology, Jagiellonian University, Poland

## Abstract

Influenza-like illness (ILI) surveillance is important to identify circulating and emerging/reemerging strains and unusual epidemiological trends. The present study aimed to give an accurate picture of the 2012–2013 ILI outbreak in Corsica by combining data from several surveillance systems: general practice, emergency general practice, hospital emergency units, intensive care units, and nursing homes. Twenty-eight respiratory viruses were retrospectively investigated from patients in general practice with ILI. Sequence analysis of the genetic changes in the hemagglutinin gene of influenza viruses (A(H1N1)pdm2009, A(H3N2) and B) was performed. The trends in ILI/influenza consultation rates and the relative illness ratios (RIRs) of having an ILI consultation were estimated by age group for the different surveillance systems analyzed. Of the 182 ILI patients enrolled by general practitioners, 57.7% tested positive for influenza viruses. Phylogenetic analyses suggested a genetic drift for influenza B and A(H3N2) viruses. The ILI/influenza surveillance systems showed similar trends and were well correlated. In accordance with virological data, the RIRs of having an ILI consultation were highest among the young (<15 years old) and decreased with age. No clusters of acute respiratory illness were declared by the sentinel nursing homes. This study is noteworthy in that it is the first extensive description of the 2012–2013 ILI outbreak in Corsica as monitored through several surveillance systems. To improve ILI surveillance in Corsica, a consortium that links together the complementary regional surveillance ILI systems described here is being implemented.

## Introduction

The continuous circulation of influenza viruses in animal species carries the potential to cause severe human illness through cross-species transmission [1]. It is also possible that virus adaptation to enable efficient and sustainable human-to-human transmission could possibly lead to worldwide pandemics [2]–[3]. Past episodes have confirmed the need for supporting influenza surveillance networks throughout the world [4]–[5].

The island of Corsica, a land bridge between Europe and Africa, is an important crossing area for infectious agents, which increases the public health importance of establishing an efficient and reliable surveillance system in the region.

In Corsica, the surveillance of disease, including influenza-like illness (ILI), is performed by several sentinel systems that are based on the activity of different health services: general practice, emergency general practice, hospital emergency units, intensive care units (ICUs), and nursing homes [6]–[8].

ILI outbreaks have been described previously using virological and epidemiological data collected by general practitioners (GPs) of the *Sentinelles* network [9]–[10]. The objective of this study was to describe the 2012–2013 ILI outbreak by combining the data from several surveillance systems that exist in Corsica. The goal was to provide useful information on the 2012–2013 ILI outbreak in Corsica and to improve the overall coordination of the ILI surveillance system for subsequent ILI outbreaks.

## Methods

### Ethics

The protocol was conducted in accordance with the Helsinki Declaration. All samples were coded and tested anonymously. None of the authors collected samples. Samples were collected and sent to authors by GPs involved in the virological surveillance. Patient information was stored according to national regulations, and access to such data was restricted (permission CNIL 471393). The patients' identities were not disclosed at any stage. Oral consent was obtained from patients by the physician investigators. For children under the age of 18, parents or legal guardians gave permission for their participation in surveillance. Consent from the child was also obtained, depending on her or his age and maturity. Based on French national laws (Law 1121-1-1° R. 1121-2), ethics committee approval and the written informed consent of patients are not required for specific microbiological diagnostic testing of the patients and further characterization of the infecting viruses.

### The Corsican *Sentinelles* Network

**Figure 1:**
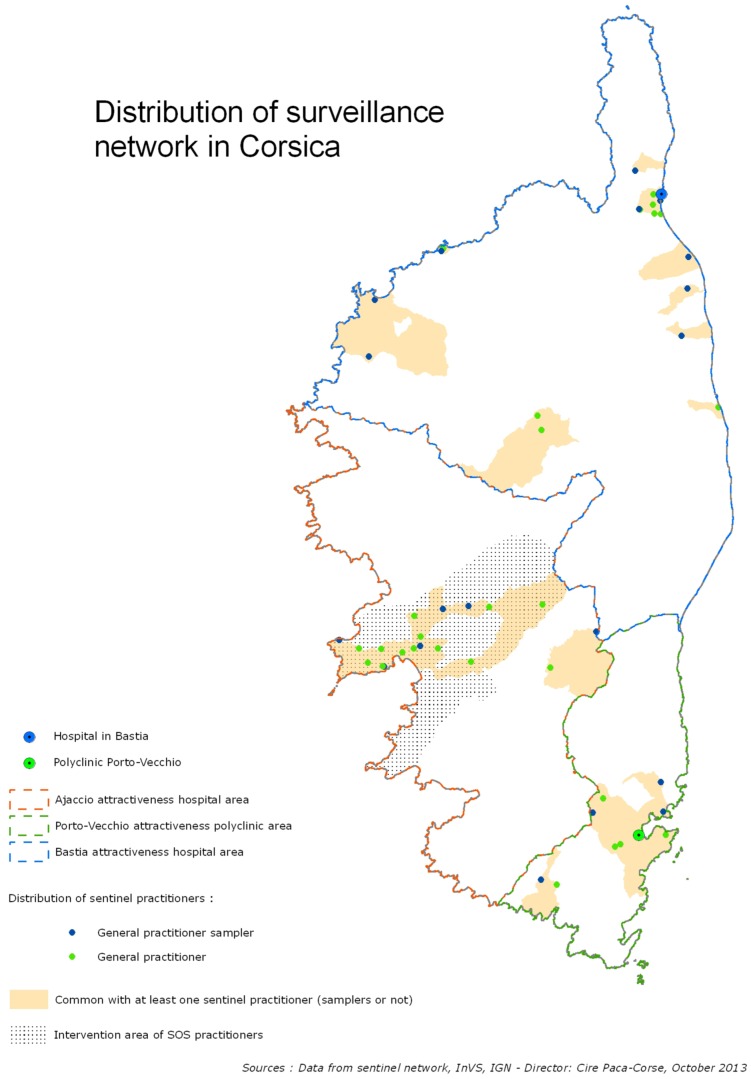
Distribution of influenza-like illness surveillance networks in Corsica.

The surveillance of ILIs is conducted in Corsica by five systems, described in detail below (Figure 1), the first of which is the Corsican *Sentinelles* network. The GPs of the Corsican *Sentinelles* network participate in the nationwide continuous surveillance of the French *Sentinelles* network by reporting each week the number of new consultations for several health indicators, including ILIs [11]–[12]. This system has been strengthened in Corsica since 2006–2007, and it currently includes 47 GPs throughout the island (16% of Corsican GPs). Of these, 19 (40.4%) participated in the 2012–2013 virological surveillance of ILIs [9].

The Corsican *Sentinelles* network was the only network performing viral testing of ILIs in Corsica. All the participating GPs received swabs and other study materials and were asked to enroll the first two patients of any age consulting a *Sentinelles* GP for an ILI in each week. The *Sentinelles* network case definition used for the inclusion of ILI patients was a sudden fever of 39°C or above, and respiratory symptoms and myalgia [13] less than 48 hours after the onset of the symptoms. Sample collection from patients with ILI was performed with Σ-Virocult® swabs (ELITech, France). The nasopharyngeal swabs were sent by mail to the virological laboratory in 1 mL of viral transport medium and stored at −80°C. Patient information, including demographic characteristics, symptoms, risk factors for severe influenza, treatment, vaccination status, and hospitalization, was documented in case report forms. All samples were tested for influenza viruses A (A(H3N2) and A(H1N1)pdm2009) and B by influenza real-time reverse transcription–polymerase chain reaction (RT–PCR) (Table 1) [9]–[14]. Samples were also tested for the simultaneous detection of 26 types/subtypes of respiratory viral pathogens using a Magicplex™ RV Panel Real-Time Test (Seegene) and as listed in Table 1. Human rhinovirus (HRV) A/B/C, human respiratory syncytial virus A/B, and human metapneumovirus results were confirmed by a supplementary real-time RT–PCR [15]–[16].

**Table 1:** Viral etiology of patients with influenza-like illness enrolled by the Corsican *Sentinelles* network.

Respiratory viruses	Total % of 182
Tested positive for at least one virus	80.8
Single infection	71.5
Co-infections	9.3
**Influenza viruses in N patients**	
All Influenza viruses	57.7
Influenza A* †	27.5
*Influenza A(H1N1)pdm2009*	18.1
*Influenza A(H3N2)*	7.1
Influenza B*	30.2
**Other respiratory viruses in N patients**	
Human Respiratory Syncytial virus (HRSV) A/B*	11.5
Human Rhinovirus (HRV) A/B/C/Human Enterovirus*	6.6
Human Adenovirus (HAdV) (A/B/C/D/E/F)*	6.0
Human Coronavirus (HCoV) 229E/NL63/OC43*	4.0
Human Bocavirus (HBoV) 1/2/3/4*	2.2
Human Metapneumovirus (HMPV)*	1.6
Human ParaInfluenzae (HPIV)1/2/3/4*	0.5

*Viruses detected by the Magiplex™ RV Panel Real-time test.

† Four influenza A viruses have not been sub-typed.

### Nucleotide Sequencing and Phylogenetic Analysis of Influenza Viruses

The hemagglutinin (HA) sequences were amplified by RT–PCR using primer sets for human A(H3N2) (nucleic acids 48–1642), A(H1N1)pdm2009 (nucleic acids 73–1200), and B (nucleic acids 1–1742) influenza viruses [9],[17],[18]. Double-stranded sequencing of the purified PCR products (primer sequences are available on request) was performed using an Applied Biosystems Sequencer (ABI 3700, Perkin-Elmer). Phylogenetic trees were constructed using a Neighborg-Joining method based on Kimura's two-parameter genetic distances matrix with 1,000 bootstrap replicates (n = 1000) using the MEGA 5.0 program [19]. The nucleotide sequence data from this study were deposited into GenBank at the National Center for Biotechnology Information (NCBI) (http://www.ncbi.nlm.nih.gov) under the following accession numbers: KF928708–KF928726 for influenza B, KF928727–KF928744 for influenza A(H1N1)pdm2009, and KC814184–KC814214 for influenza A(H3N2).

### The Emergency General Practice Network: *SOS Médecins*

The French Institute for Public Health Surveillance (*Institut de Veille Sanitaire*, InVS) developed in 2003 a syndromic surveillance system based on the activity of emergency health professionals through an organization called *SOS Médecins*, which is the first emergency network of GPs and health care in France [8]. In Corsica, this emergency general practice surveillance system was implemented in December 2010. It is based on the activity of 11 GPs covering a population of 100 000 habitants (the Ajaccio area in the southwest of Corsica; see Figure 1) and responding to private house calls 24 h a day, 7 days a week [8]. The GPs report on a daily basis the sociodemographic and medical data for each patient visited (age, sex, diagnosis and hospitalization) [20]. For maximal comparability with respect to the definition of the French *Sentinelles* network for the inclusion of ILI patients, the *SOS Médecins* ILI definition (sudden fever of 38.5°C or above, respiratory symptoms and myalgia) is coded according to the International Classification of Primary Care (code “R80 Influenza/R80 ILI) [20]. The weekly number of visits coded as “R80 Influenza/R80 ILI” by *SOS Médecins*, was analyzed. The chosen indicator of ILI activity was the number of ILIs coded per week divided by the total number of visits coded per week.

### Hospital Emergency Units: The OSCOUR Network

In Corsica, the OSCOUR network has been based since 2011–2012 on the activity of the two hospitals located in the northeast (Bastia) and southeast (Porto-Vecchio) of Corsica (Figure 1). Data (age, sex, diagnosis and hospitalization) were collected directly from outpatients' computerized medical files completed during medical consultations and transmitted encrypted to the InVS 7 days a week. Information about patients with a diagnosis of influenza (*International* Classification of *Diseases*-10: J09 to J11) was extracted weekly. The J09 code corresponds to influenza due to A(H1N1)pdm2009, J10 corresponds to influenza caused by other identified influenza viruses, and J11 corresponds to an ILI caused by unidentified influenza viruses. The indicator chosen was the number of cases coded J09 to J11 per week divided by the total number of visits coded per week.

### Surveillance of Severe Influenza Cases in Intensive Care Units (ICUs)

Clinicians are required to report all probable and confirmed influenza cases admitted to an ICU to the regional office of the InVS (*cellules de l'InVS en region*), using a standardized notification form. Patient information, including demographic characteristics, symptoms, risk factors for severe influenza, treatment, vaccination status, and hospitalization was collected. Confirmed cases were defined as patients who tested positive for influenza by RT–PCR performed on a nasal swab.

### Clusters of Acute Respiratory Infections (ARIs) in Nursing Homes

A surveillance system in nursing homes for the elderly has been implemented in Corsica since 2011–2012, to assist them in managing clusters of ARIs. A cluster is defined as five cases or more within 4 days and must be reported to the local health authority using a standardized notification form. An evaluation survey was conducted in July–September 2013, after the 2012–2013 ILI outbreak, providing figures about vaccination coverage (VC) against influenza for residents and for health care workers.

### Statistical Analyses

The total study population was grouped into the following six age groups: 0–4, 5–14, 15–29, 30–44, 45–64, and >65 years old. These were chosen based on standard clinical groupings according to probable influenza exposure/risk environments. For continuous variables, groups were compared by Student's *t*-tests or Mann–Whitney tests as appropriate. The chi-squared or Fisher's exact test (as appropriate) was used to compare dichotomous variables between groups. The demographic data on the Corsican population were provided by the French National Institute of Statistic and Economic Studies [21].

The age-specific burden of illness was assessed using the relative illness ratio (RIR) [22]. This ratio divides the contribution of a specific age group *i* to the total number of ILI cases 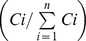 by its proportion of the general population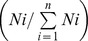:
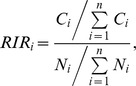
where *C_i_* is the number of ILI cases in age group *i* (there are *n* age groups in total), and *N_i_* is the total population of an age group *i*. *RIR_i_* helps to assess the under- or overrepresentation of age group *i* among ILI cases: a ratio above 1 indicates disproportionate risk. Confidence intervals (CIs) were estimated with the Poisson exact method [23]. Spearman rank correlation coefficients (*ρ*) of ILI time series were determined for the epidemic period among the surveillance networks analyzed. Analyses were performed using STATA software (v. 11.0, StataCorp LP, Texas, USA) and R software (v. 2.13.2) (http://www.r-project.org) [24]. The epidemiological forms used to collect the data from ILI patients consulting the *Sentinelles* GPs were entered anonymously in EpiData (v. 1.4.2 (http://www.epidata.dk).

## Results

### Trends in ILI/Influenza

**Figure 2:**
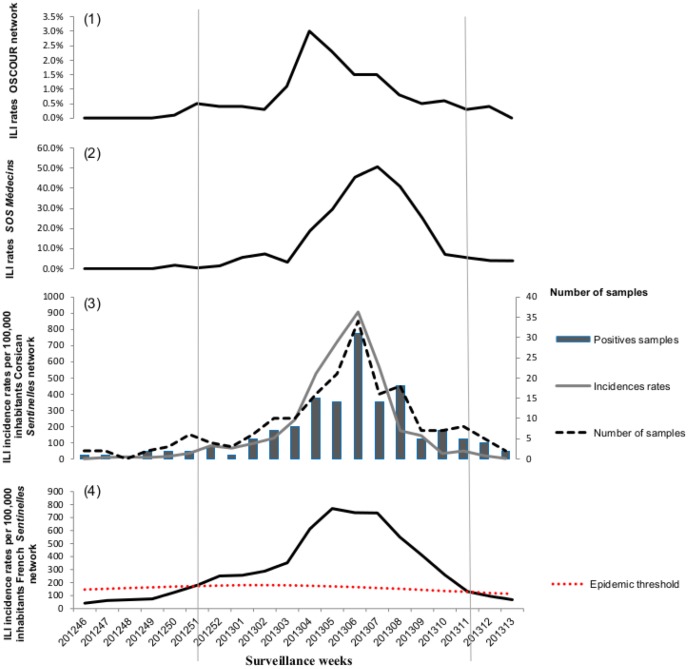
Temporal distribution of influenza-like illness (ILI) consultation rates from November 2012 (week 46) to April 2013 (week 14) by surveillance networks in Corsica. From top to bottom, (**1**) Weekly rates (black line) of J09 to J11 diagnostics estimated over the totality of number of visits coded per week at hospital emergencies (OSCOUR network), (**2**) Weekly rates (black line) of R80 Influenza/R80 ILI diagnostics estimated over the totality of number of visits coded per week by GPs of the emergency general practice network (*SOS Médecins*). (**3**) ILI incidence rates (grey line) per 100 000 inhabitants and weekly distribution of ILI samples positives (histogram bars) to at least one respiratory viruses analyzed (GPs of the Corsican *Sentinelles* network). (**4**) ILI incidence rates (black line) per 100 000 inhabitants (GPs of the French *Sentinelles* network) and epidemic threshold (red dotted line) calculated by a periodic regression on model applied to the former observed data (Serfling'method) [44].

The ILI consultation rates are reported in Figure 2. The French *Sentinelles* network's ILI rates indicate that the 2012–2013 epidemic in mainland France started during week 51, 2012 (from December 17 to December 23, 2012), peaked at week 5, 2013 (from January 28 to February 3, 2013), and fell below the epidemic threshold during week 11, 2013 (from March 11 to March 17, 2013). All surveillance systems analyzed had a significant and strong correlation with the ILI consultation rates of the Corsican *Sentinelles* GPs (Figure 2) during the epidemic period. The highest correlation coefficient was seen for the incidence reported by the French *Sentinelles* network (*ρ* = 0.862; *p*<0.05), followed by the ILI rates estimated by emergency GPs in Corsica (*SOS Médecins*) (*ρ* = 0.821; *p*<0.05) and hospital emergency units (OSCOUR) (*ρ* = 0.615; *p*<0.05) (Figure 2).

### Relative Illness Ratio (RIR)

**Figure 3:**
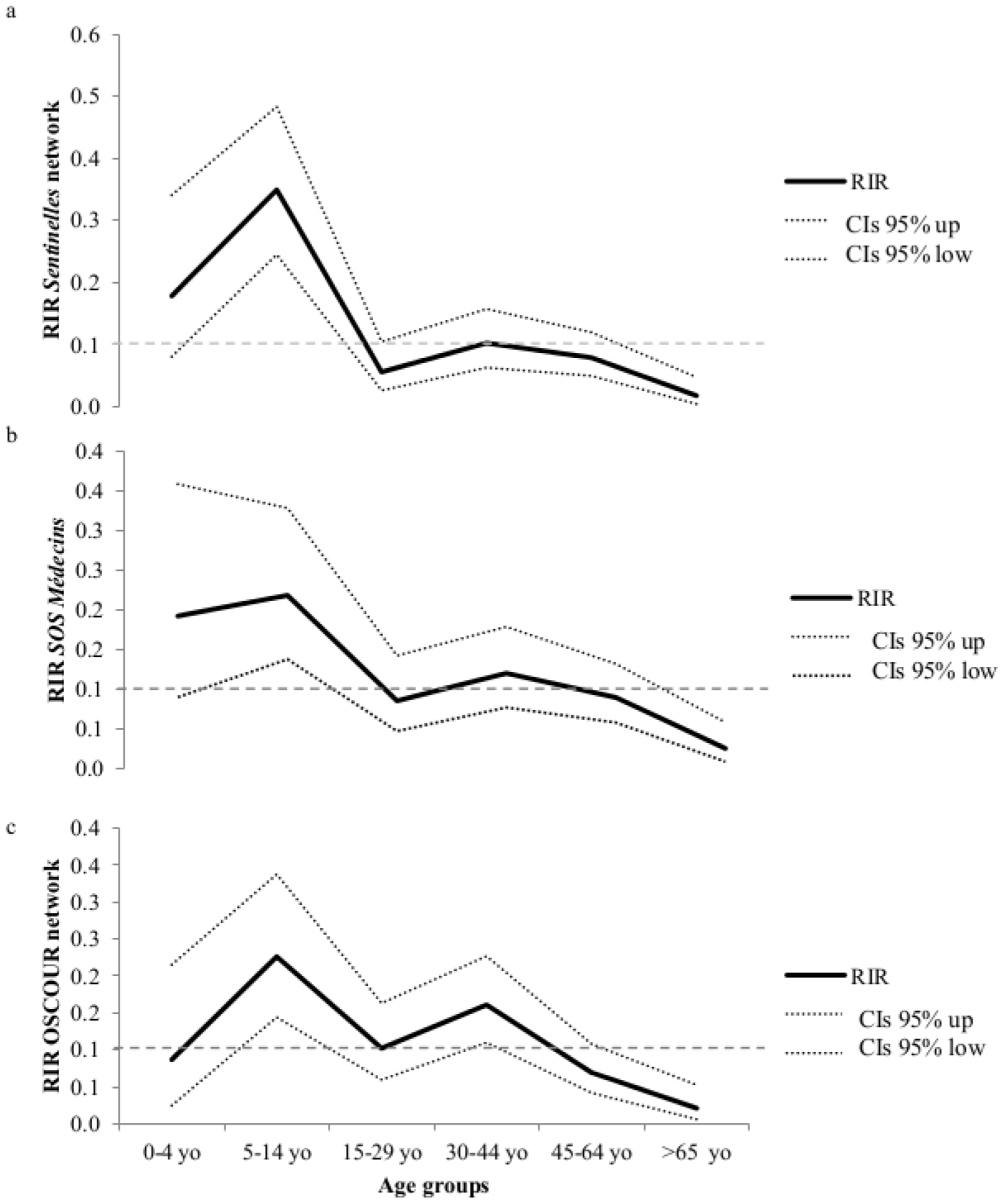
Relative Illness Ratio (RIR) by age-group for the 2012–2013 epidemic (black line) and 95% confidence intervals (CIs) (dotted line) by surveillance ILI network: (a) Corsican *Sentinelles* network; (b) *SOS Médecins* and (c) OSCOUR network.

The RIRs of ILI consultations by age group and surveillance system during the influenza epidemic period are shown in Figure 3. Across all surveillance networks, the RIRs were consistently highest for the youngest patients (<15 years old) compared with the adults. The RIRs peaked for school-aged children (5–14 years old), were closer to unity for young adults (30–44 years old), and decreased for adults over 45. The lowest RIRs were observed for patients aged over 65 years.

### Number of Severe Influenza Cases in ICUs

Three severe influenza cases were reported by ICU clinicians. They median patient age was 62 years (min = 59, max = 66). The patients had at least one risk factor for severe influenza (obesity, pulmonary pathology, or aged >65 years, respectively) and had not been vaccinated with the 2012–2013 seasonal vaccine. Two had laboratory-confirmed A(H1N1)pdm2009.

### Surveillance of Outbreaks in Nursing Homes

Sixty-one percent (16/26) of nursing homes participated in the surveillance. They were representative of all nursing homes in terms of their number of beds (*p-value* = 0.91), departments (*p-value* = 1) and their public/private status (*p-value* = 0.60). No clusters of ARIs were declared during the 2012–2013 ILI outbreak and epidemic period by the 16 participating nursing homes. The global VC of residents against seasonal 2012–2013 influenza virus was 87.6% (95% CI, (86.3%–89.0%)). For the health care workers, the global VC was 16.0% (95% CI, (13.0%–18.9%)).

### Viral Etiology

One hundred eighty-two nasopharyngeal samples were collected by 19 GPs of the Corsica *Sentinelles* network. Patients' characteristics are reported in Table 2. The median age of the 182 patients reported by the virology surveillance system was 30.0 years (min = 1, max = 73), and 59.3% were female. Six percent of ILI patients had been vaccinated with the 2012–2013 seasonal trivalent influenza vaccine (data not shown in Table 2).

**Table 2:** Demographics of patients with influenza-like illness (ILI) enrolled by the Corsican *Sentinelles* network.

Characteristics	All ILI patients N = 182	Positives to at least one respiratory virus N = 147	Positives to one Influenza viruses N = 87	Positives to one respiratory viruses N = 43	Coinfections N = 17
**Demographic**					
Age -median	30 [1–73]	29 [1–73]	32 [1–73]	18 [1–73]	16 [2]–[41]
Female sex (%)	59.3	40.8	61.0	55.8	58.8
**Age groups (%)**					
0–4	12.1	15.0	15.0	14.0	17.6
5–14	18.1	14.3	15.0	30.2	29.4
15–29	18.7	16.3	14.0	16.3	29.4
30–44	26.7	23.8	30.0	11.6	23.5
45–64	18.7	19.1	24.0	16.3	0
>65	5.5	4.7	22.0	11.6	0

Of the 182 samples (Table 1), 80.8% were positive for at least one virus. Single infections accounted for 71.5% of patients. Influenza viruses were detected in 57.7% of the patients. Influenza B was detected in 30.2% of the ILI patients. Influenza A was detected in 27.5% of the patients, of whom 18.1% had A(H1N1)pdm2009. Coinfections were detected in 9.3% of the ILI patients. The most frequent combination was an influenza B virus with one other respiratory pathogen (Table 3). Patients with no infection, a single infection, or coinfection did not differ with regard to sex or clinical symptoms. The distribution of the respiratory viruses among the different age groups is presented in Table 4. The rate of positivity for at least one respiratory virus decreased significantly (*p* = 0.012). Coinfections were only detected in patients less than 45 years old.

**Table 3:** Coinfections of respiratory viruses in patients with influenza-like illness patients enrolled by the Corsican *Sentinelles* network.

Co-infections	N
Influenza B, Influenza A	1
Influenza B, HAdV	3
Influenza B, HBoV	2
Influenza B, HRV	1
Influenza B, HRSV	2
Influenza B, A(H1N1)pdm2009	1
A (H1N1)pdm2009, HAdV	1
A (H1N1)pdm2009, HBoV	2
A (H1N1)pdm2009,HMPV	1
A (H1N1)pdm2009, HRSV	1
A(H3N2), HRSV	1
HRSV, HAdV	1
**Total N (% of 182)**	**17 of 182 (9.3%)**

**Table 4:** Distribution of viral etiologies according to age group in patients with influenza-like illness enrolled by the Corsican *Sentinelles* network.

Age groups (%)	0–4 N = 22	5–14 N = 33	15–29 N = 34	30–44 N = 49	45–64 N = 34	>65 N = 10	Pvalue
**Positive (any type)**	100	93.9	70.6	71.4	82.4	70.0	0.012
**At least one Influenza virus**	72.7	51.5	50.0	61.2	61.8	20.0	0.090
**Influenza A**	40.9	18.8	29.4	30.6	23.5	10.0	0.385
**Influenza B**	36.4	30.3	20.6	32.6	38.2	10.0	0.431
**At least one other viruses than Influenza**	36.4	54.5	35.3	16.3	20.6	50.0	0.004
**Coinfections**	13.6	15.2	14.7	8.2	0.0	0.0	0.122
**HRSV (A/B)**	9.1	24.2	14.7	2.0	14.7	0.0	0.040
**HRV**	13.6	9.1	5.8	0.0	0.0	40.0	0.000

### Phylogenetic Analyses of Influenza Viruses

**Figure 4:**
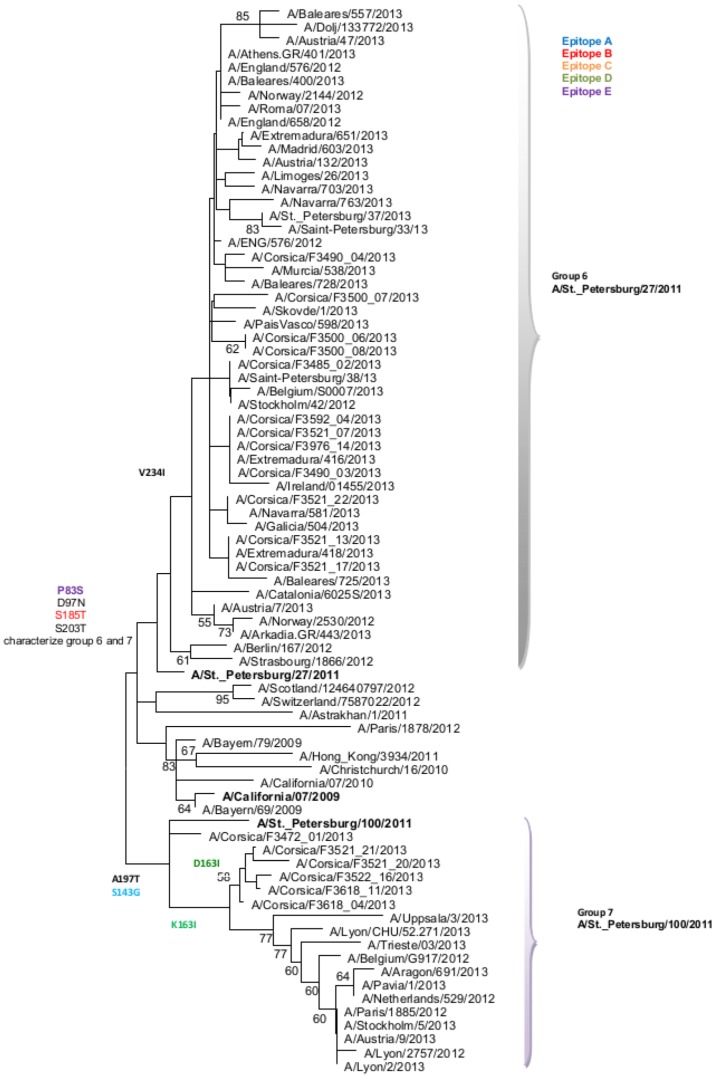
Evolutionary relationship of A(H1N1)pdm2009 influenza viruses sampled in Corsica during the 2012–2013 influenza like-illness outbreak. The evolutionary history was inferred using the Neighbor-Joining method. The bootstrap consensus tree inferred from 1000 replicates is taken to represent the evolutionary history of the taxa analyzed. Branches corresponding to partitions reproduced in less than 50% bootstrap replicates are collapsed. The analysis involved 79 sequences.

Among the 33 samples of A(H1N1)pdm2009 influenza virus detected in 2012–2013, 54.5% (N = 18) were randomly selected, and their HA sequences were compared with those of vaccine and reference strains for phylogenetic analysis (Figure 4; Table S1). The A(H1N1)pdm2009 viruses clustered within genetic groups 6 and 7, which were characterized by the D97N and S185T (antigenic site B) mutations with respect to A/California/7/2009 (2012–2013 vaccine influenza strain). Group 6 was characterized by the additional mutation D163I (antigenic site D), and group 7 by the mutations S143G (antigenic site A) and A197T, with respect to A/California/7/2009.

**Figure 5:**
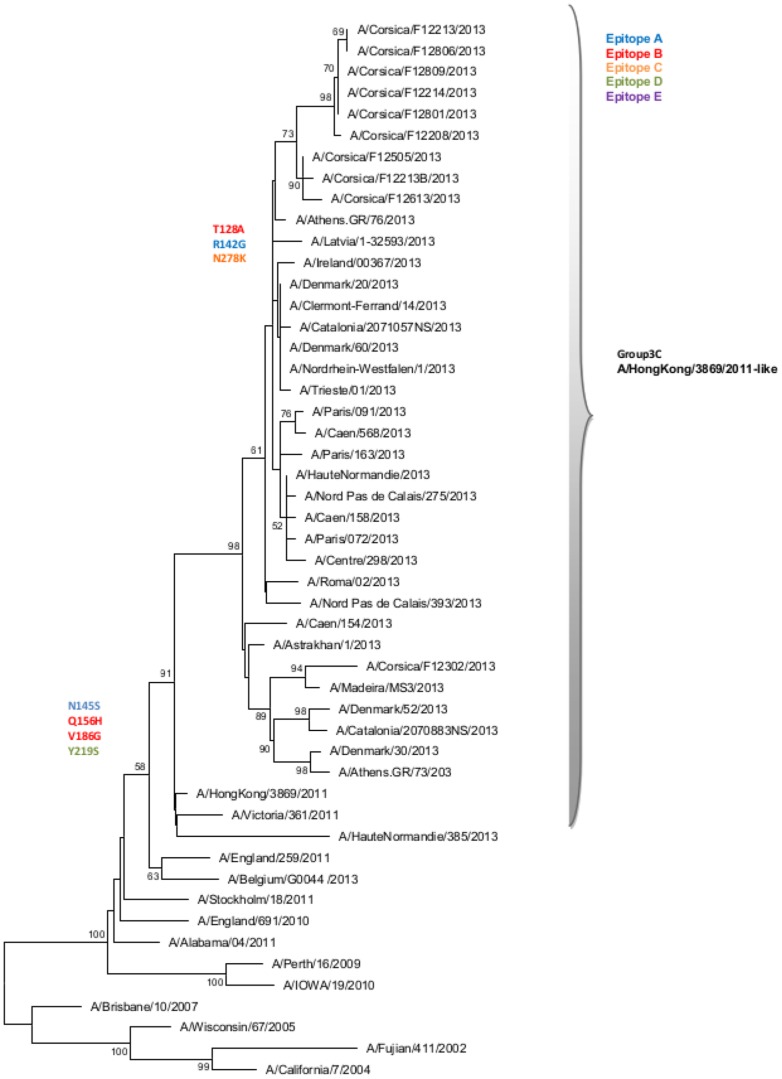
Evolutionary relationship of A(H3N2) influenza viruses sampled in Corsica during the 2012–2013 influenza like-illness outbreak. The evolutionary history was inferred using the Neighbor-Joining method. The bootstrap consensus tree inferred from 1000 replicates is taken to represent the evolutionary history of the taxa analyzed. Branches corresponding to partitions reproduced in less than 50% bootstrap replicates are collapsed. The analysis involved 50 sequences.

The HA of 10 of 13 (77.0%) randomly selected Corsican A(H3N2) influenza strains obtained during the 2012–2013 influenza outbreak clustered in the A/Hong Kong/3869/2011-like group 3C (Figure 5; Table S2). The group 3C strains are characterized by the mutations T128A (antigenic site B), R142G (antigenic site A), and N278K (antigenic site C) with respect to A/Victoria/361/2011 (reference vaccine strain for 2012–2013).

**Figure 6:**
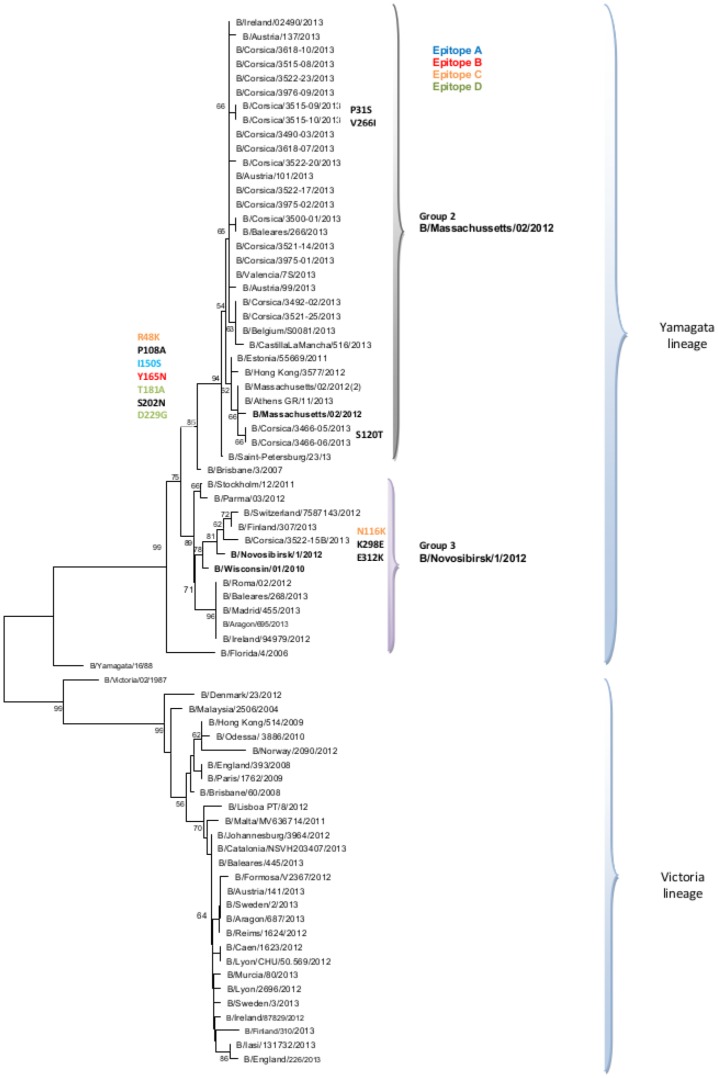
Evolutionary relationship of B influenza viruses sampled in Corsica during the 2012–2013 influenza like-illness outbreak. The evolutionary history was inferred using the Neighbor-Joining method. The bootstrap consensus tree inferred from 1000 replicates is taken to represent the evolutionary history of the taxa analyzed. Branches corresponding to partitions reproduced in less than 50% bootstrap replicates are collapsed. The analysis involved 75 sequences.

The HA of 19 of 55 (34.5%) randomly selected influenza B viruses detected in 2012–2013 was compared with vaccine and reference strains for phylogenetic analysis (Figure 6; Table S3). All influenza B viruses isolated in Corsica during 2012–2013 were of the Yamagata lineage and fell into genetic group 2 (95%; N = 18) and group 3 (5%; N = 1). The strains belonging to group 2 were characterized by the mutations R48K (antigenic site C), P108A and I150S (antigenic site A), Y165N (antigenic site B), T181A (antigenic site D), and D229G (antigenic site D) with respect to the B/Wisconsin/1/2010 vaccine strain of the 2012–2013 season. The influenza B strain belonging to group 3 was characterized by the mutations N116K (antigenic site C), K298E, and E312K.

## Discussion

The ILI consultation rates reported by the *Sentinelles* network remain the basis of surveillance in Corsica, because the system integrates epidemiological and virological information as well as data about the molecular characterization of the influenza viruses. All surveillance systems analyzed showed a strong correlation with the ILI consultation rates declared by the Corsican *Sentinelles* network. Overall, the RIRs of consulting different medical services that participated in ILI surveillance (*Sentinelles* network, *SOS Médecins*, or OSCOUR) showed similar trends, which decreased with age. Similarly, virological data from the ILI patients enrolled from the GPs of the *Sentinelles* network confirmed this trend, as the rate of positivity for at least one respiratory virus decreased significantly with age. No clusters of ARIs were declared during the 2012–2013 ILI outbreak by the sentinel nursing homes.

Among the ILI patients enrolled by the *Sentinelles* GPs, 80.8% tested positive for at least one of the respiratory pathogens analyzed, which is a higher positivity rate than in previous reports [2],[25],[26]. This result could be explained by the high specificity of the ILI case definition used in the present study [13] and the unusual length of the 2012–2013 ILI outbreak (13 weeks) [27]. In this study, coinfections were detected in persons aged 44 years and younger at a rate of 9.3%, consistent with previous studies [26], [28], [29]. Several studies have reported an association between viral coinfections and increased morbidity, but these remains controversial [30]. In our study, all coinfections were associated with mild ILI disease.

In the present study, the rate of positivity for at least one respiratory virus decreased significantly with age. It should be noted that while human respiratory syncytial virus (A/B), was the second most frequently detected respiratory virus after influenza in patients aged less than 15 years, in agreement with previous studies [31], HRV was the most frequently detected virus in patients aged >65 years. Information about the non influenza viral infections such as HRV to ILI episodes in older persons is poor, partly because of difficulties in diagnosis related to atypical presentations and low viral loads [32]–[34]. Previously, it has been suggested that HRV could be the cause of exacerbations of chronic respiratory diseases and could increase morbidity rates in frail persons as, including the elderly [33]–[34]. Overall, the RIR of consulting for an ILI was similar among the different surveillance networks analyzed here and decreased significantly with age. Two age groups were characterized by an RIR above 1: preschool and school-aged children less than 14 years old, and working adults aged 30–44 years. This peak in the 30–44-year-old group could be explained by their proximity to children as parents. This trend is in accordance with the viral etiology of the epidemic, characterized by the cocirculation of A(H1N1)pdm2009 and B influenza viruses [35].

In Corsica, no clusters of ARIs were declared during the 2012–2013 ILI outbreak in sentinel nursing homes. This was remarkable because of the significant cocirculation of influenza A and B viruses and of other respiratory viruses. This finding is likely attributable to the high VC against influenza viruses among residents, similar to the VC previously reported in French nursing homes [36], and a certain degree of cross-protection from previous exposure to influenza A(H1N1)pdm2009 [37]. It could also be due to the result of better general hygiene management, as demonstrated by the rarity of clusters of gastroenteritis (N = 3) declared by the same sentinel nursing homes. However, it is unlikely to have arisen through a good VC among staff, as this was low (16%), similar to staff VC levels previously reported that ranged from 10% to 50% [36].

During the 2012–2013 ILI outbreak, epidemiological studies of laboratory-confirmed cases estimated a low-to-moderate vaccine effectiveness against influenza viruses [38]–[41]. Phylogenetic analyses of the HA gene in Corsica showed that all influenza B viruses belonged to the Yamagata lineage. Except for one strain that belonged to the group 3, all other Influenza B strains fell into genetic group 2 and were characterized by five mutations compared with the vaccine strain, located at four antigenic sites, which could suggest a drift [42]. The A(H1N1)pdm2009 viruses detected in Corsica belonged to groups 6 and 7, and showed a good match with the vaccine strain [43]. As previously reported, the HA sequences of A(H3N2) viruses isolated in Corsica during the 2012–2013 season [10] were characterized by seven amino acid substitutions at four antigenic sites compared with A/Victoria/361/2011 (2012–2013 vaccine strain), suggesting a drift [42].

Several limitations were inherent in the surveillance systems examined in this study. The monitoring of ILI, is mainly based on clinical surveillance, as the virological surveillance, for instance, was carried out by the *Sentinelles* network. The lack of virological confirmation for the patients of the emergency general practice network (*SOS Médecins*), who visited the hospital emergency departments (OSCOUR) and/or in nursing homes is an important gap. The evaluation of the causative agents of ILI in patients enrolled by the GPs of the *Sentinelles* network did not cover all possible respiratory pathogens, as other bacterial or viral pathogens that can cause ILI were not investigated. It is possible that a low viral titer could not be detected by RT-–PCR. Moreover, antigenic characterization of the influenza viruses that were characterized genetically was not performed. Overall, the patients were enrolled using a rather specific ILI case definition (especially for the *Sentinelles* network and for the *SOS Médecins* network), excluding infected patients with atypical ILI symptom presentations, including older persons, as well as asymptomatic infections. Finally, the epidemic threshold was only available for the ILI consultation rates reported by the *Sentinelles* GPs at a national level, which may not be adequate at a regional level with respect to the GPs *Sentinelles* network and with respect to the other local surveillance systems.

To improve ILI surveillance in Corsica, a consortium that links together the complementary regional surveillance ILI systems described here is being implemented. Today, the Corsican *Sentinelles* network is the only network performing viral testing of ILIs in Corsica. This consortium will allow the integration of the virological surveillance of more than 20 respiratory viruses with the ILI clinical surveillance of the OSCOUR network (hospital emergency units), the *SOS Médecins* network (emergency network of GPs), and the surveillance of ARIs in the sentinel nursing homes. The integration of virological surveillance in these systems will permit the acquisition of essential information on the spread of the analyzed respiratory viruses in the island, especially influenza viruses, and about the severity of the influenza epidemics.

This study is noteworthy in that it is the first extensive description of the 2012–2013 ILI outbreak and the influenza epidemic in Corsica using several surveillance systems. Overall, data collected from the different surveillance systems were well correlated, and they suggest that the influenza epidemic had a low impact on older persons.

## Supporting Information

Table S1 Amino acid substitutions observed in antigenic sites (A–E) of the hemagglutinin protein of 19 A(H1N1)2009 influenza viruses isolated between November 2012 to April 2013 in Corsica Island, France.
(DOCX)

Table S2 Amino acid substitutions observed in antigenic sites (A–E) of the hemagglutinin protein of 10 A(H3N2) influenza viruses isolated between November and April 2013 in Corsica Island, France.
(DOCX)

Table S3 Amino acid substitutions observed in antigenic sites (A–E) of the hemagglutinin protein of 19 B influenza viruses isolated between November 2012 and April 2013 in Corsica Island, France.
(DOCX)
