# Correction: Epidemiology and Viral Etiology of the Influenza-Like Illness in Corsica during the 2012–2013 Winter: An Analysis of Several Sentinel Surveillance Systems

**DOI:** 10.1371/journal.pone.0107844

**Published:** 2014-09-08

**Authors:** The PLOS ONE Staff

There are errors in affiliations 4 and 5 for Cécile Souty. Affiliation 4 should be: Sorbonne Universités, UPMC Univ Paris 06, UMR_S 1136, F-75013, Paris, France. Affiliation 5 should be: INSERM, UMR_S 1136, F-75013, Paris, France.

The affiliations for the first author are incorrect. Laëtitia Minodier is also affiliated with #5 INSERM, UMR_S 1136, F-75013, Paris, France.
