# Supplementary material for: Epidemiology and Viral Etiology of the Influenza-Like Illness in Corsica during the 2012–2013 Winter: An Analysis of Several Sentinel Surveillance Systems
Source: PLoS One. 2014 Jun 24;9(6):e100388. doi: 10.1371/journal.pone.0100388 (PMC4069071; doi:10.1371/journal.pone.0100388)
Supplement: Table S1 — Amino acid substitutions observed in antigenic sites (A–E) of the hemagglutinin protein of 19 A(H1N1)2009 influenza viruses isolated between November 2012 to April 2013 in Corsica Island, France. (DOCX) [file pone.0100388.s001.docx]

**Epidemiology and Viral Etiology of the Influenza-like Illness in Corsica During the 2012–2013 Winter: An Analysis of Several Sentinel Surveillance Systems.**

Laëtitia Minodier^1^*****, Christophe Arena ^1,2^, Guillaume Heuze ^3^, Marc Ruello ^3^, Jean Pierre Amoros ^1^, Cécile Souty ^4,5^, Laurent Varesi^1^, Alessandra Falchi ^1^

1. *EA7310, Laboratoire de Virologie, Université de Corse-Inserm, France*
2. *Observatoire régional de la Santé de Corse, France*
3. *Cellule de l’InVS en région, Ajaccio, France*
4. *Sorbonne Universités, UPMC Univ Paris 06, UMRS 1136, Institut Pierre Louis d’Epidémiologie et de Santé Publique, Paris, France*
5. *Inserm, UMRS 1136, Institut Pierre Louis d’Epidémiologie et de Santé Publique, Paris, France*

**Supplementary information files**

**Table S1**: Amino acid substitutions observed in antigenic sites (A-E) of the hemagglutinin protein of 19 A(H1N1)2009 influenza viruses isolated between November 2012 to April 2013 in Corsica Island, France

| Antigenic sites | **83** | **84** | **94** | **97** | **125** | **134** | **141** | **143** | **160** | **163** | **175** | **183** | **185** | **197** | **203** | **205** | **216** | **222** | **223** | **234** | **249** | **250** | **256** |
| --- | --- | --- | --- | --- | --- | --- | --- | --- | --- | --- | --- | --- | --- | --- | --- | --- | --- | --- | --- | --- | --- | --- | --- |
|  | E | E | D |  | B | A | A | A | B | D |  | B | B |  |  | D | D | D | D |  |  |  |  |
| **A/California/07/2009** | P | S | D | D | N | A | A | S | K | K | V | S | S | A | S | R | I | D | Q | V | V | V | A |
| A/Astrakhan/1/2011 | S | . | . | N | . | . | . | . | . | . | . | . | . | . | T | K | V | . | . | . | L | . | . |
| A/Hong_Kong/3934/2011 | **S** | **.** | **.** | **.** | **.** | **T** | **S** | **.** | **.** | **.** | **.** | **P** | **.** | **.** | **T** | **.** | **.** | **N** | **.** | **.** | **.** | **.** | **.** |
| A/Christchurch/16/2010 | **S** | **.** | **N** | **.** | **D** | **.** | **.** | **.** | **T** | **.** | **.** | **.** | **.** | **.** | **T** | **.** | **.** | **N** | **.** | **.** | **.** | **A** | **.** |
| **A/St._Petersburg/27/2011** | S | . | . | N | . | . | . | . | . | . | . | . | T | . | T | . | . | . | R | . | . | . | . |
| A/Corsica/F3521_17/2013 | S | . | . | N | . | . | . | . | . | . | . | . | T | . | T | . | . | . | . | I | . | . | . |
| A/Corsica/F3521_22/2013 | S | . | . | N | . | . | . | . | . | . | . | . | T | . | T | . | . | . | . | I | . | . | . |
| A/Corsica/F3592_04/2013 | S | . | . | N | . | . | . | . | . | . | . | . | T | . | T | . | . | . | . | I | . | . | . |
| A/Corsica/F3976_14/2013 | S | . | . | N | . | . | . | . | . | . | . | . | T | . | T | . | . | . | . | I | . | . | . |
| A/Corsica/F3485_02/2013 | S | . | . | N | . | . | . | . | . | . | . | . | T | . | T | . | . | . | . | I | . | . | . |
| A/Corsica/F3490_03/2013 | S | . | . | N | . | . | . | . | . | . | . | . | T | . | T | . | . | . | . | I | . | . | . |
| A/Corsica/F3500_06/2013 | S | . | . | N | . | . | . | . | . | . | . | . | T | . | T | . | . | . | . | I | . | . | . |
| A/Corsica/F3500_08/2013 | S | . | . | N | . | . | . | . | . | . | . | . | T | . | T | . | . | . | . | I | . | . | . |
| A/Corsica/F3521_07/2013 | S | . | . | N | . | . | . | . | . | . | . | . | T | . | T | . | . | . | . | I | . | . | . |
| A/Corsica/F3521_13/2013 | S | . | . | N | . | . | . | . | . | . | . | . | T | . | T | . | . | . | . | I | . | . | . |
| A/Corsica/F3472_01/2013 | S | . | . | N | . | . | . | . | . | . | E | . | T | T | T | . | . | . | . | . | . | . | . |
| A/Corsica/F3490_04/2013 | S | . | . | N | . | . | . | . | . | . | . | . | T | . | T | . | . | . | . | I | . | . | . |
| A/Corsica/F3500_07/2013 | S | . | . | N | . | . | . | . | . | . | . | . | T | . | T | . | . | . | . | I | . | . | . |
| **A/St._Petersburg/100/2011** | **S** | **.** | **.** | **.** | **.** | **.** | **.** | **G** | **.** | **.** | **.** | **.** | **T** | **T** | **T** | **.** | **.** | **.** | **R** | **.** | **.** | **.** | **.** |
| A/Corsica/F3521_21/2013 | S | . | . | N | . | . | . | G | . | I | . | . | T | T | T | . | . | . | . | . | . | . | . |
| A/Corsica/F3521_20/2013 | S | G | . | N | . | . | . | G | . | I | . | . | T | T | T | . | . | . | . | . | . | . | . |
| A/Corsica/F3522_16/2013 | S | . | . | N | . | . | . | G | . | I | . | . | T | T | T | K | . | . | . | . | . | . | . |
| A/Corsica/F3618_04/2013 | S | . | . | N | . | . | . | G | . | I | . | . | T | T | T | . | . | . | . | . | . | . | . |
| A/Corsica/F3618_11/2013 | S | . | . | N | . | . | . | G | . | I | . | . | T | T | T | K | . | . | . | . | . | . | . |
| A/Corsica/F3472_01/2013 | S | . | . | N | . | . | . | . | . | . | E | . | T | T | T | . | . | . | . | . | . | . | . |
