# Supplementary material for: Epidemiology and Viral Etiology of the Influenza-Like Illness in Corsica during the 2012–2013 Winter: An Analysis of Several Sentinel Surveillance Systems
Source: PLoS One. 2014 Jun 24;9(6):e100388. doi: 10.1371/journal.pone.0100388 (PMC4069071; doi:10.1371/journal.pone.0100388)
Supplement: Table S2 — Amino acid substitutions observed in antigenic sites (A–E) of the hemagglutinin protein of 10 A(H3N2) influenza viruses isolated between November and April 2013 in Corsica Island, France. (DOCX) [file pone.0100388.s002.docx]

**Epidemiology and Viral Etiology of the Influenza-like Illness in Corsica During the 2012–2013 Winter: An Analysis of Several Sentinel Surveillance Systems.**

Laëtitia Minodier^1^*****, Christophe Arena ^1,2^, Guillaume Heuze ^3^, Marc Ruello ^3^, Jean Pierre Amoros ^1^, Cécile Souty ^4,5^, Laurent Varesi^1^, Alessandra Falchi ^1^

1. *EA7310, Laboratoire de Virologie, Université de Corse-Inserm, France*
2. *Observatoire régional de la Santé de Corse, France*
3. *Cellule de l’InVS en région, Ajaccio, France*
4. *Sorbonne Universités, UPMC Univ Paris 06, UMRS 1136, Institut Pierre Louis d’Epidémiologie et de Santé Publique, Paris, France*
5. *Inserm, UMRS 1136, Institut Pierre Louis d’Epidémiologie et de Santé Publique, Paris, France*

**Supplementary information files**

**Table S2**: Amino acid substitutions observed in antigenic sites (A-E) of the hemagglutinin protein of 10 A(H3N2) influenza viruses isolated between November and April 2013 in Corsica Island, France.

| **Antigenic sites** | **48** | **62** | **81** | **121** | **124** | **128** | **140** | **141** | **142** | **144** | **145** | **150** | **156** | **158** | **159** | **172** | **173** | **183** | **186** | **188** | **189** | **193** | **194** | **196** | **198** | **212** | **219** | **223** | **278** | **312** |
| --- | --- | --- | --- | --- | --- | --- | --- | --- | --- | --- | --- | --- | --- | --- | --- | --- | --- | --- | --- | --- | --- | --- | --- | --- | --- | --- | --- | --- | --- | --- |
|  | E | E | E |  | A | B | A | A | A | A | A |  | B | B | B | D | D |  | B | B | B | B | D | B | B |  | D | D | C | C |
| A/Victoria/361/2011 | I | E | N | N | S | T | I | R | R | N | N | R | Q | N | F | E | Q | H | V | D | K | F | L | A | S | A | Y | I | N | S |
| **A/Perth/16/2009** | **T** | **.** | **.** | **.** | **.** | **.** | **.** | **.** | **.** | **.** | **.** | **.** | **.** | **.** | **.** | **.** | **.** | **.** | **.** | **.** | **.** | **.** | **.** | **.** | **A** | **.** | **.** | **V** | **.** | **N** |
| A/Fujian/411/2002 | T | . | . | . | . | . | K | . | . | . | K | . | H | K | Y | . | K | L | G | . | S | S | . | . | A | T | S | V | . | N |
| A/California/7/2004 | T | . | . | . | . | . | K | . | . | . | . | . | H | K | . | . | K | . | G | N | N | S | . | T | A | T | S | V | . | N |
| A/Alabama/04/2011 | T | . | . | . | . | . | . | . | . | . | . | . | H | . | . | . | . | . | G | . | . | . | . | . | A | . | S | V | . | N |
| A/Wisconsin/67/2005 | T | . | . | . | . | . | K | . | . | . | . | . | H | K | . | . | K | . | . | . | N | . | . | . | A | T | S | . | . | N |
| A/Brisbane/10/2007 | T | . | . | . | . | . | . | . | . | . | . | . | H | K | . | . | K | . | G | . | N | . | P | . | A | T | S | V | . | N |
| A/IOWA/19/2010 | T | . | . | . | . | . | . | . | . | . | . | . | H | . | . | . | . | . | S | . | . | . | . | T | A | . | S | V | . | N |
| A/England/691/2010 | A | . | . | . | . | . | . | . | . | . | . | . | H | . | . | . | . | . | G | . | . | . | . | . | A | . | S | V | . | . |
| **A/Stockholm/18/2011** | **T** | **.** | **.** | **.** | **.** | **.** | **.** | **.** | **.** | **D** | **S** | **.** | **H** | **.** | **.** | **.** | **.** | **.** | **G** | **.** | **.** | **.** | **.** | **.** | **A** | **.** | **S** | **.** | **.** | **N** |
| **A/England/259/2011** | **A** | **.** | **.** | **.** | **.** | **.** | **.** | **.** | **.** | **.** | **S** | **.** | **H** | **.** | **.** | **.** | **.** | **.** | **G** | **.** | **.** | **.** | **.** | **.** | **.** | **.** | **F** | **.** | **.** | **.** |
| **A/HongKong/3869/2011** | **.** | **.** | **.** | **.** | **.** | **.** | **.** | **.** | **.** | **.** | **.** | **.** | **H** | **.** | **.** | **.** | **.** | **.** | **G** | **.** | **.** | **.** | **.** | **.** | **.** | **.** | **S** | **.** | **.** | **.** |
| A/Corsica/F12213B/2013 | . | . | . | . | . | A | . | . | G | . | S | . | H | . | . | . | . | . | G | . | . | . | . | . | . | . | S | . | K | . |
| A/Corsica/F12505/2013 | . | . | . | . | . | A | . | . | G | . | S | . | H | . | . | . | . | . | G | . | . | . | . | . | . | . | S | . | K | . |
| A/Corsica/F12613/2013 | . | . | . | . | . | A | . | . | G | . | S | . | H | . | . | . | . | . | G | . | . | . | . | . | . | . | S | . | K | . |
| A/Corsica/F12214/2013 | . | . | . | . | . | A | . | . | G | . | S | . | H | . | . | . | . | . | G | . | . | . | . | . | . | . | S | . | K | . |
| A/Corsica/F12801/2013 | . | . | . | . | . | A | . | . | G | . | S | . | H | . | . | . | . | . | G | . | . | . | . | . | . | . | S | . | K | . |
| A/Corsica/F12809/2013 | . | . | . | . | . | A | . | . | G | . | S | . | H | . | . | . | . | . | G | . | . | . | . | . | . | . | S | . | K | . |
| A/Corsica/F12208/2013 | . | . | . | . | . | A | . | . | G | . | S | . | H | . | . | . | . | . | G | . | . | . | . | . | . | . | S | . | K | . |
| A/Corsica/F12213/2013 | . | . | . | . | . | A | . | . | G | . | S | . | H | . | . | . | . | . | G | . | . | . | . | . | . | . | S | . | K | . |
| A/Corsica/F12806/2013 | . | . | . | . | . | A | . | . | G | . | S | . | H | . | . | . | . | . | G | . | . | . | . | . | . | . | S | . | K | . |
| A/Corsica/F12302/2013 | . | . | . | . | . | . | . | . | . | . | S | . | H | . | . | . | . | . | G | . | . | . | . | . | . | . | S | . | K | . |
