# Supplementary material for: Epidemiology and Viral Etiology of the Influenza-Like Illness in Corsica during the 2012–2013 Winter: An Analysis of Several Sentinel Surveillance Systems
Source: PLoS One. 2014 Jun 24;9(6):e100388. doi: 10.1371/journal.pone.0100388 (PMC4069071; doi:10.1371/journal.pone.0100388)
Supplement: Table S3 — Amino acid substitutions observed in antigenic sites (A–E) of the hemagglutinin protein of 19 B influenza viruses isolated between November 2012 and April 2013 in Corsica Island, France. (DOCX) [file pone.0100388.s003.docx]

**Epidemiology and Viral Etiology of the Influenza-like Illness in Corsica During the 2012–2013 Winter: An Analysis of Several Sentinel Surveillance Systems.**

Laëtitia Minodier^1^*****, Christophe Arena ^1,2^, Guillaume Heuze ^3^, Marc Ruello ^3^, Jean Pierre Amoros ^1^, Cécile Souty ^4,5^, Laurent Varesi^1^, Alessandra Falchi ^1^

1. *EA7310, Laboratoire de Virologie, Université de Corse-Inserm, France*
2. *Observatoire régional de la Santé de Corse, France*
3. *Cellule de l’InVS en région, Ajaccio, France*
4. *Sorbonne Universités, UPMC Univ Paris 06, UMRS 1136, Institut Pierre Louis d’Epidémiologie et de Santé Publique, Paris, France*
5. *Inserm, UMRS 1136, Institut Pierre Louis d’Epidémiologie et de Santé Publique, Paris, France*

**Supplementary information files**

**Table S3**: Amino acid substitutions observed in antigenic sites (A-E) of the hemagglutinin protein of 19 B influenza viruses isolated between November 2012 and April 2013 in Corsica Island, France.

| **Antigenic Sites** | **29** | **31** | **48** | **88** | **108** | **116** | **120** | **150** | **165** | **173** | **181** | **202** | **206** | **224** | **229** | **234** | **251** | **266** | **298** | **312** | **344** |
| --- | --- | --- | --- | --- | --- | --- | --- | --- | --- | --- | --- | --- | --- | --- | --- | --- | --- | --- | --- | --- | --- |
|  |  |  | C |  |  | C |  | A | B |  | D |  | B |  | D |  |  |  |  |  |  |
| **B/Wisconsin/01/2010** | V | P | R | R | P | N | S | I | Y | L | T | S | D | V | D | T | M | V | K | E | K |
| B/Brisbane/3/2007 | . | . | K | . | A | . | . | S | N | . | . | N | . | . | G | . | . | . | . | . | . |
| B/Massachusetts/02/2012 | . | . | K | . | A | . | . | S | N | . | A | N | . | . | G | . | . | . | . | . | . |
| B/Stockholm/12/2011 | A | . | . | . | . | . | . | . | . | Q | . | N | . | . | . | . | V | . | . | . | . |
| B/Florida/4/2006 | . | . | . | K | . | . | . | S | N | . | . | N | . | . | S | . | . | . | . | . | . |
| B/Hong_Kong/3577/2012 | . | . | K | . | A | . | . | S | N | . | A | N | . | . | G | . | . | . | . | . | . |
| **B/Massachusetts/02/2012** | . | . | K | . | A | . | . | S | N | . | A | N | . | . | G | . | . | . | . | . | . |
| B/Corsica/3466-05/2013 | . | . | K | . | A | . | T | S | N | . | A | N | . | . | G | . | . | . | . | . | . |
| B/Corsica/3466-06/2013 | . | . | K | . | A | . | T | S | N | . | A | N | . | . | G | . | . | . | . | . | . |
| B/Corsica/3490-03/2013 | . | . | K | . | A | . | . | S | N | . | A | N | . | . | G | . | . | . | . | . | . |
| B/Corsica/3492-02/2013 | . | . | K | . | A | . | . | S | N | . | A | N | . | D | G | . | . | . | . | . | . |
| B/Corsica/3500-01/2013 | . | . | K | . | A | . | . | S | N | . | A | N | . | . | . | . | . | . | . | . | . |
| B/Corsica/3515-08/2013 | . | . | K | . | A | . | . | S | N | . | A | N | . | . | G | . | . | . | . | . | . |
| B/Corsica/3515-09/2013 | . | S | K | . | A | . | . | S | N | . | A | I | . | . | G | . | . | I | . | . | . |
| B/Corsica/3515-10/2013 | . | S | K | . | A | . | . | S | N | . | A | I | . | . | G | . | . | I | . | . | . |
| B/Corsica/3521-14/2013 | . | . | K | . | A | . | . | S | N | . | A | N | . | . | G | . | . | . | . | . | . |
| B/Corsica/3521-25/2013 | . | . | K | . | A | . | . | S | N | . | A | N | . | . | G | . | . | . | . | . | . |
| B/Corsica/3522-17/2013 | . | . | K | . | A | . | . | S | N | . | A | N | E | . | G | . | . | . | . | . | . |
| B/Corsica/3522-20/2013 | . | . | K | . | A | . | . | S | N | . | A | N | . | . | G | . | . | . | . | . | . |
| B/Corsica/3522-23/2013 | . | . | K | . | A | . | . | S | N | . | A | N | . | . | G | . | . | . | . | . | . |
| B/Corsica/3618-07/2013 | . | . | K | . | A | . | . | S | N | . | A | N | . | . | . | . | . | . | . | . | . |
| B/Corsica/3618-10/2013 | . | . | K | . | A | . | . | S | N | . | A | N | . | . | G | K | . | . | . | . | . |
| B/Corsica/3975-01/2013 | . | . | K | . | A | . | . | S | N | . | A | N | . | . | G | . | . | . | . | . | . |
| B/Corsica/3975-02/2013 | . | . | K | . | A | . | . | S | N | . | A | N | . | . | G | . | . | . | . | . | . |
| B/Corsica/3976-09/2013 | . | . | K | . | A | . | . | S | N | . | A | N | . | . | G | . | . | . | . | . | T |
| **B/Novosibirsk/1/2012** | . | . | . | . | . | K | . | . | . | . | . | . | . | . | . | . | . | . | E | K | . |
| B/Corsica/3522-15b/2013 | . | . | . | . | . | K | . | . | . | . | . | . | . | . | . | . | . | . | E | K | . |
